# Learning curve of robot-assisted total knee arthroplasty and its effects on implant position in asian patients: a prospective study

**DOI:** 10.1186/s12891-023-06422-w

**Published:** 2023-04-27

**Authors:** Ho Jung Jung, Min Wook Kang, Jong Hwa Lee, Joong Il Kim

**Affiliations:** Department of Orthopedic Surgery, Kangnam Sacred Heart Hospital, Hallym University College of Medicine, Seoul, Korea

**Keywords:** Learning curve, Lower limb alignment, Operative time, Total knee arthroplasty, Robotic surgery

## Abstract

**Background:**

Robot-assisted total knee arthroplasty (r-TKA) can reportedly achieve more accurate implant positioning than conventional total knee arthroplasty (c-TKA), although its learning curve is controversial. Moreover, few studies have investigated r-TKA in Asians, who have different anatomical characteristics. This study aimed to determine the learning curve for r-TKA and compare implant positions between r-TKA and c-TKA according to the learning curve in Asian patients.

**Methods:**

This prospective study included 50 consecutive c-TKAs (group C), followed by 50 consecutive r-TKAs conducted using the MAKO robotic system (Stryker, USA). Cumulative summation analyses were performed to assess the learning curve for operative time in r-TKA. Accordingly, the r-TKA cases were divided into the initial (group I) and proficiency cases (group P). The femoral and tibial component positions in the coronal, sagittal, and axial planes and lower limb alignment were compared among the three groups.

**Results:**

r-TKA was associated with a learning curve for operative time in 18 cases. The operative time was significantly shorter in groups C and P than that in group I, with no significant difference between groups C and P. Groups I and P demonstrated fewer outliers with respect to lower limb alignment, femoral component coronal position, axial position, and tibial component sagittal position than those in group C, with no significant difference between groups I and P.

**Conclusion:**

The operative time did not differ significantly between r-TKA and c-TKA after the learning curve. Surgeons could expect more accurate and reproducible lower limb alignment and implant positioning with r-TKA in Asian patients, irrespective of the learning curve.

## Background

Knee osteoarthritis (OA) can cause progressive pain and dysfunction, eventually leading to a decline in the quality of life. Total knee arthroplasty (TKA) is considered the most effective treatment for pain relief and functional recovery in patients with severe knee OA [1, 2]. However, there are conflicting reports regarding patient dissatisfaction even after TKA [3, 4]. Although the causes of dissatisfaction are numerous, improper positioning of the prosthesis due to inaccurate bone resection could be a major reason. Numerous studies have reported that component malpositioning can lead to residual pain, instability, and functional deterioration, which eventually affects the longevity of the prosthesis [5–9]. Therefore, accurate prosthesis positioning is essential for improving clinical outcomes and implant survival after TKA [10, 11].

In conventional TKA (c-TKA), the incidence of outliers exceeding 3° from the planned lower limb alignment or implant position has been reported to be up to 30% [12]. Robot-assisted total knee arthroplasty (r-TKA) has recently gained popularity in the field of arthroplasty to reduce these outliers. Several studies have reported that r-TKA demonstrated better radiological and clinical outcomes compared to c-TKA with respect to lower limb alignment, knee joint stability, functional recovery, length of hospital stay, and prosthesis survivorship, although there are some concerns regarding the learning curve [13–16]. However, these findings are not fully applicable to Asian patients because previous studies only focused on Western populations. Asian patients have different body features and anatomical characteristics, such as a higher incidence of constitutional varus deformity, anterior and lateral femoral bowing, and proximal tibia varus compared to their Western counterparts, which could affect not only the learning curve of r-TKA, but also lower limb alignment and implant position after TKA. However, studies evaluating the learning curve in r-TKA or comparing the lower limb alignment and implant position between r-TKA and c-TKA in Asian patients are lacking.

Therefore, this study aimed to determine the learning curve for r-TKA and compare the lower limb alignment and implant position between r-TKA and c-TKA according to the learning curve of r-TKA in Asian patients. We hypothesized that accurate lower limb alignment and implant position in Asian patients may be better achieved with r-TKA than those with c-TKA, irrespective of the stages in the learning curve.

## Materials and methods

### Patients

**Fig. 1:**
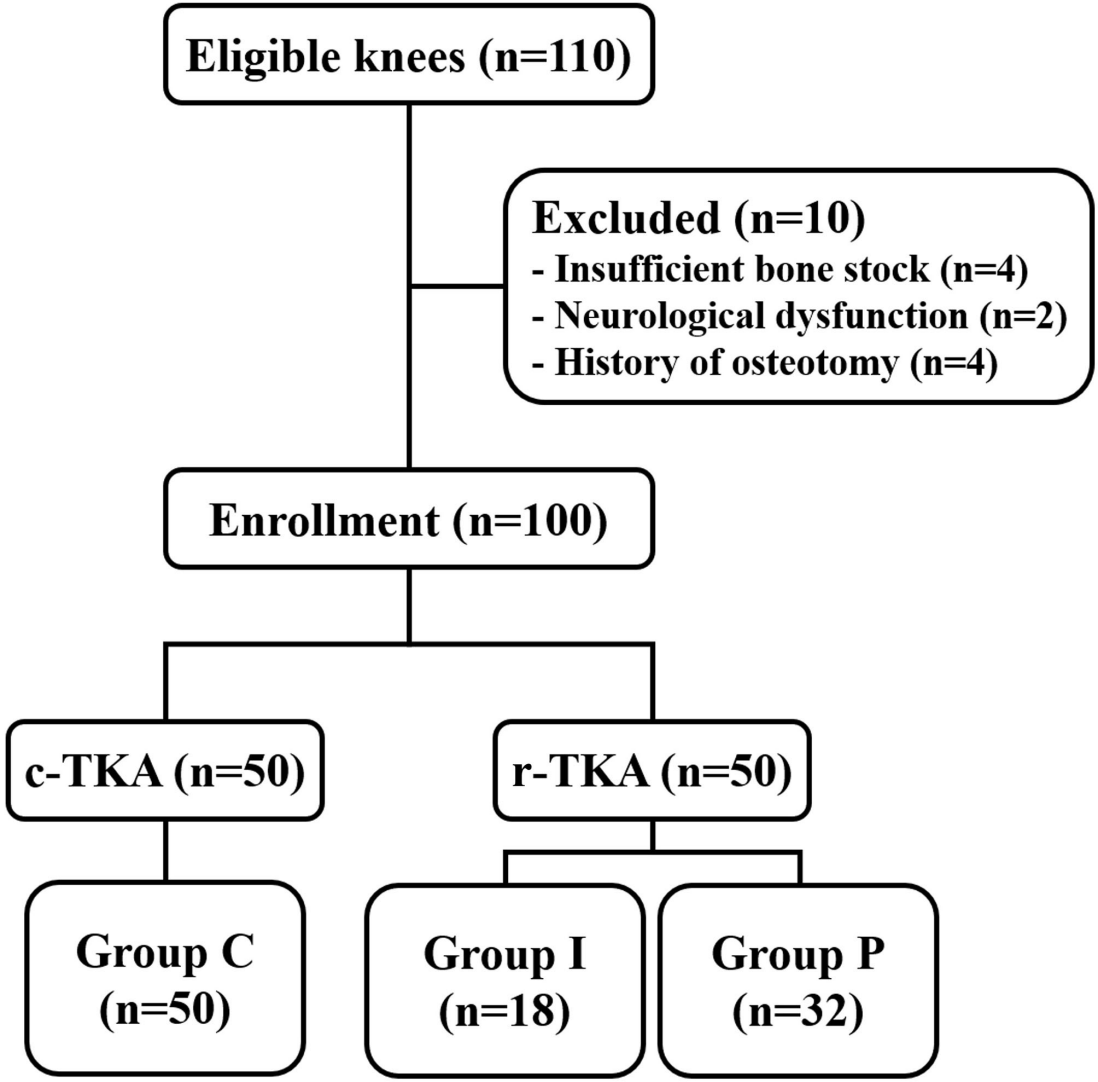
Flow diagram of patient enrollment ^*^ c-TKA, conventional total knee arthroplasty; r-TKA, robot-assisted total knee arthroplasty; group C, conventional total knee arthroplasty group; group I, initial phase group; group P, proficiency phase group

This prospective study was conducted at a single center between August 2021 and June 2022. A total of 94 patients with 110 knees who experienced failure of conservative treatment for knee OA (Kellgren–Lawrence grades III–IV) and decided to undergo TKA were offered enrollment. The exclusion criteria were as follows: insufficient bone stock that needed an augmentation block or long stem, the presence of neurological dysfunction that limited the standing position, and a history of osteotomy with the affected knee. After study participants provided informed consent, 84 patients with 100 knees were finally included in the study, according to the above-mentioned criteria. The patients were assigned to different treatment groups according to the date of surgery. The first 50 consecutive knees underwent c-TKA (group C); thereafter, 50 consecutive knees underwent r-TKA after installation of the MAKO Robotic Arm Interactive Orthopedic System (RIO; Stryker, Kalamazoo, MI, USA) in December 2021. Based on the inflection point of the learning curve [17, 18], r-TKA cases were divided into initial cases (group I) and proficiency cases (group P) (Fig. 1). This study was approved by the Institutional Review Board of Hallym University Kangnam Sacred Heart Hospital.

### Surgical technique and rehabilitation

All surgical procedures were conducted by a single high-volume (> 200 cases per year) surgeon (KJI) specializing in knee arthroplasty without previous navigation or robotic surgery experience. Mandatory theoretical and one cadaveric training were performed with MAKO RIO® for 4 h prior to the commencement of the study. All patients in both groups underwent treatment with an identical surgical protocol, except for the MAKO RIO® in the r-TKA group. A posterior stabilizing prosthetic (Triathlon®, Stryker, Kalamazoo, MI, USA) was implanted with patellar resurfacing using a standard medial parapatellar approach and tourniquet inflation (300 mmHg).

In the case of r-TKA, two pins were inserted into the femur and tibia 10 cm away from the previous skin incision. Femoral and tibial arrays were placed on the pins, and the bone surface was registered. The patient-specific computed tomography (CT)-based bone model was confirmed using the registered landmarks, followed by bone resection, which was performed based on the planned preoperative values.

For c-TKA, bone resection was performed with reference to the intramedullary (IM) guide of the distal femur and the extramedullary guide (EM) of the proximal tibia. The femoral entry point was drilled at the intersection of the IM centerline and distal cortex of the femur along the sagittal and coronal planes. The femoral component rotation was set to be parallel to the surgical transepicondylar axis (sTEA). The tibial alignment guide was positioned parallel to the longitudinal axis of the tibia in the coronal plane. Thereafter, it was adjusted to the target slope of 2° in the sagittal plane.

The tourniquet was deflated after final fixation of the cemented prosthesis, and the remaining focus of bleeding was cauterized after manual compression using gauze packing at the surgical site during cement hardening. A closed suction drain was placed in the joint, and the capsule was closed in a watertight fashion. Range of motion exercises were initiated on postoperative day (POD) 1. The drain was removed, and ambulation with a walker was initiated on POD 2.

### Radiographic evaluation

**Fig. 2:**
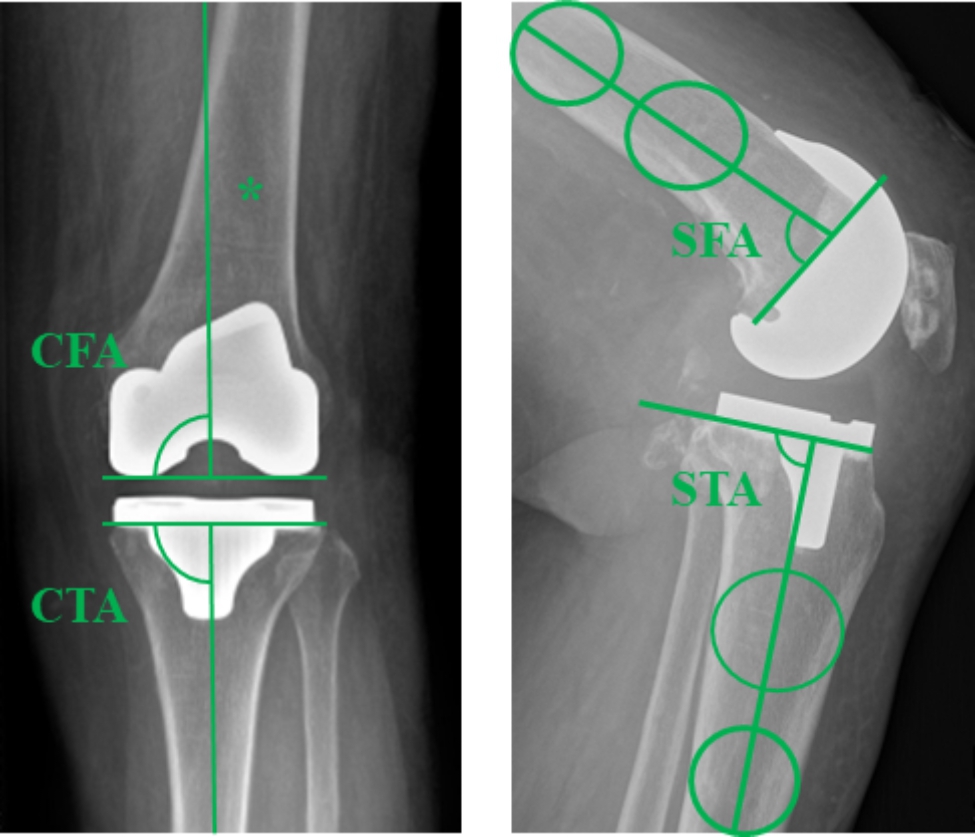
Component position angle on radiograpy. CFA was determined as the medial angle between the line connecting the femoral component condyles and the mechanical axis of the femur. CTA was defined as the medial angle between the horizontal tibial tray and the mechanical axis of the tibia. SFA was the proximal angle between the line perpendicular to the distal cement surface and the anatomical axis of the femur. STA was defined as the angle between the axis of the horizontal tibial tray and the anatomical axis of the tibia ^*^CFA, coronal femoral alignment; CTA, coronal tibial alignment; SFA, sagittal femoral alignment; STA, sagittal tibial alignment

**Fig. 3:**
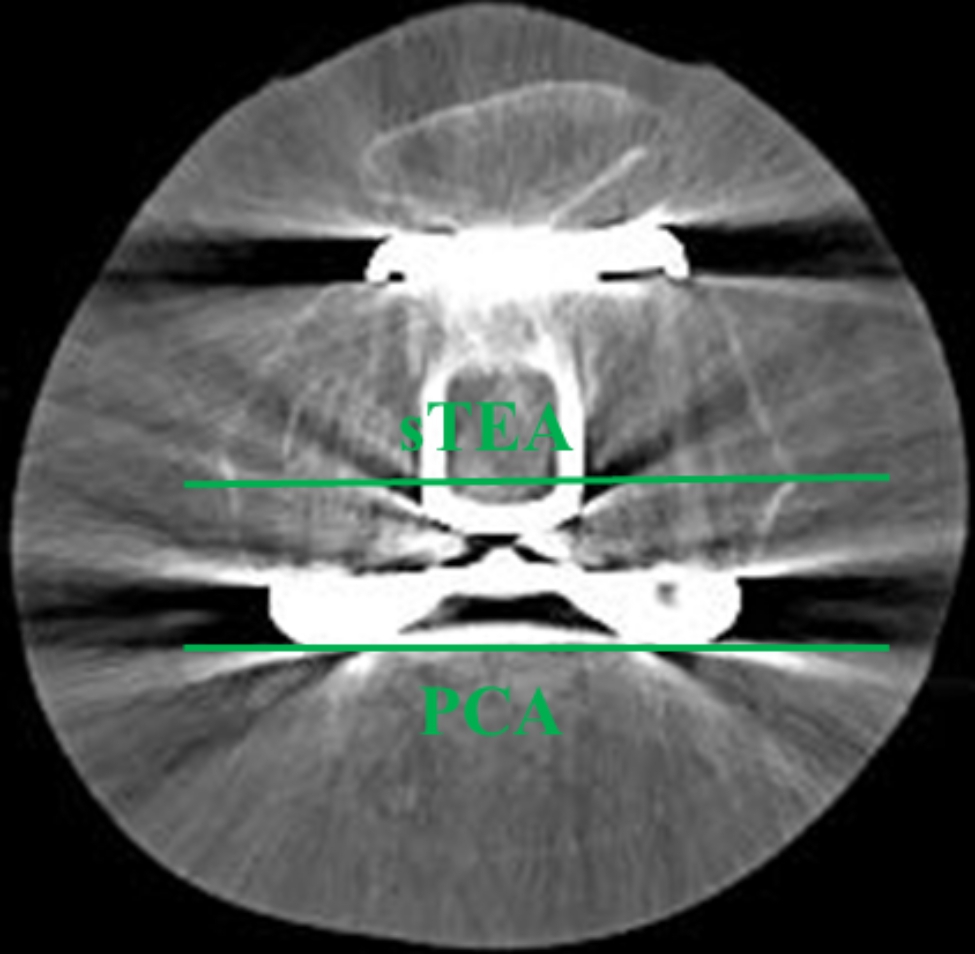
Component position angle on CT. The FCR was determined as the angle between the sTEA and PCA ^*^CT, computer tomography; FCR, femoral component rotation; sTEA, surgical transepicondylar axis; PCA, posterior condylar axis

All patients underwent a standing radiograph series on POD 6 to determine the accuracy of prosthesis positioning. The hip–knee–ankle (HKA) angle was defined as the angle formed by the mechanical axis of the femur and tibia on full-length standing anteroposterior (AP) radiographs. Alignment of the femoral and tibial components was measured in the coronal and sagittal planes. Coronal femoral alignment (CFA) was defined as the medial angle between the line connecting the femoral component condyles and the mechanical axis of the femur on full-length standing AP radiographs. Coronal tibial alignment (CTA) was defined as the medial angle between the horizontal tibial tray and the mechanical axis of the tibia on full-length standing AP radiographs. Sagittal femoral alignment (SFA) was the proximal angle between the line perpendicular to the distal cement surface and the anatomical axis of the femur on lateral standing radiographs. Sagittal tibial alignment (STA) was defined as the angle between the axis of the horizontal tibial tray and the anatomical axis of the tibia on lateral standing radiographs (Fig. 2). All patients underwent CT assessment on POD 2 to evaluate the rotational alignment of the femoral component. The angle between the sTEA and posterior condylar axis (PCA) was denoted as the femoral component rotation (FCR) (Fig. 3).

In the c-TKA group, the target alignment of CFA, CTA, and SFA was determined to be 90°, HKA was 0°, and STA was 88°. The rotational alignment of the femoral component was parallel to the surgical TEA. In the r-TKA group, all target alignments were planned according to the MAKO system using a patient-specific CT-based bone model. This procedure was optimized by assessing the implant size, limb alignment, and implant position using virtual implant templates.

The differences between the target alignment values and the true alignment values were calculated to determine the position error. The valgus of the coronal alignment was denoted as a negative value, and the varus was denoted as a positive value. Flexion of sagittal alignment was denoted as a negative value and extension as a positive value. Internal rotation of the femoral component was negative, and external rotation was positive. Difference values diverging at ± 3° were considered outliers. The operative time, which was defined as the interval between initial skin incision and wound closure, was determined from the anesthesia record.

### Statistical analysis

Sample size calculation was performed in accordance with a previous study that evaluated the outlier ratio of r-TKA compared to c-TKA. A minimum of 50 knees were required in each group to perform Fisher’s exact test with a power of 0.80 and alpha value of 0.05 [19].

Data analysis was performed using the independent *t*-test and one-way analysis of variance for continuous variables, and Pearson’s chi-square test or Fisher’s exact test for categorical variables. The learning curve of the operative time in r-TKA was determined using cumulative summation (CUSUM) analysis, according to previous studies [18, 20, 21].

Radiographic parameters were measured twice by two independent observers, with a 2-week interval between the measurements. Intra- and inter-observer measurement reliabilities were assessed using intraclass correlation coefficients. All statistical analyses were performed using SPSS Statistics (version 25.0, IBM, USA). Statistical significance was set at *p* < 0.05.

## Results

**Fig. 4:**
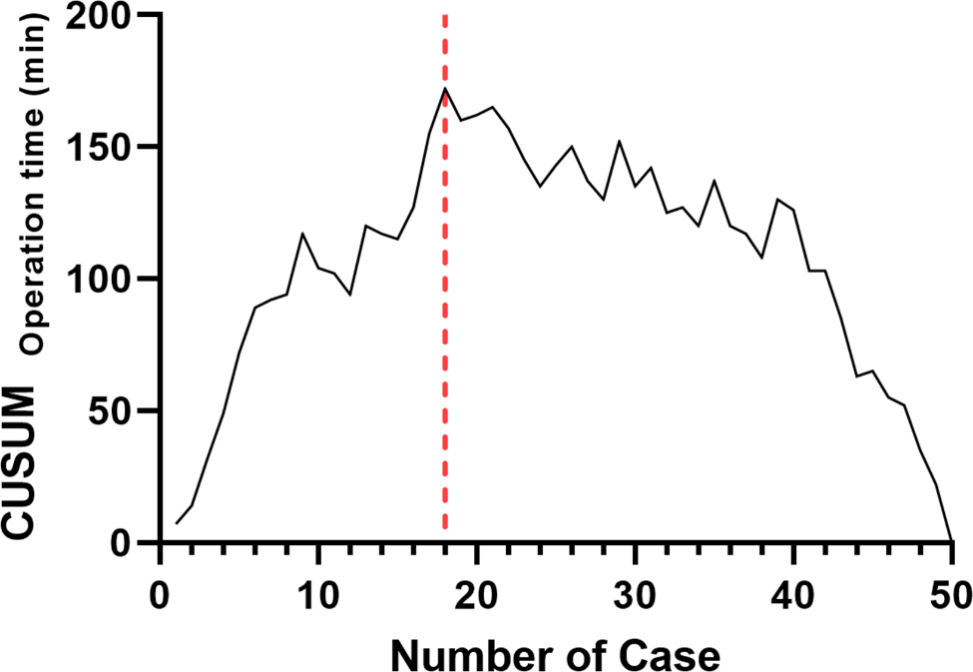
Chart of CUSUM analysis for r-TKA. r-TKA was associated with a learning curve for operative time in 18 cases. ^*^ CUSUM, cumulative summation; r-TKA, robot-assisted total knee arthroplasty

The demographic characteristics of the study population are presented in Table 1. CUSUM analysis revealed that r-TKA was associated with a learning curve for operative time in 18 cases (Fig. 4). The operative time differed significantly among the three groups; the operative time was significantly shorter in groups C and P than that in group I, with no significant difference between groups C and P (group C: 91.94 ± 12.96 min, group I: 112.75 ± 13.32 min, and group P: 98.53 ± 12.45 min; *p* < 0.001). Groups I and P demonstrated significant differences in the position error of sagittal tibial component (group C: 3.72 ± 2.42°, group I: 0.86 ± 0.82°, and group P: 0.84 ± 0.77°; *p* < 0.001) and FCR (group C: −2.11 ± 1.58°, group I: 1.62 ± 1.12°, and group P: 1.30 ± 0.83°; *p* < 0.001) than those in group C. However, no significant differences were observed in any radiographic parameters between groups I and P (Table 2).

**Table 1:** Participants’ baseline characteristics

Variable	Conventional TKA (n = 50)	Robot-arm assisted TKA (n = 50)	p-value ^b^
Age (years)	70.54 ± 9.02	70.13 ± 4.84	0.778
Sex (male/female)	8/42	7/43	0.421
BMI (kg/m^2^)	26.71 ± 3.75	26.79 ± 3.21	0.959
K–L grade (III/IV)	37:13	41:9	0.470
HKA angle (°) ^c^	6.21 ± 2.73	5.83 ± 2.69	0.764
Range of motion (°)	119.7 ± 18.83	118.4 ± 19.7	0.983

^a^ TKA, total knee arthroplasty; BMI, body mass index; K–L grade, Kellgren–Lawrence grade; HKA, hip–knee–ankle. ^b^ Independent t test for continuous variables and Pearson’s chi-square test for categorical variables. ^c^ A positive value denotes varus malalignment

**Table 2:** Comparison of position errors among the three groups

Variable	Conventional TKA (n = 50)	Robot-arm assisted TKA (n = 50)		p-value ^b^
		Initial stage	Proficiency stage	
HKA angle (°) ^c^	1.18 ± 2.06	0.87 ± 1.09	0.44 ± 1.36	0.165
Coronal femoral component (°) ^c^	0.69 ± 1.48	0.55 ± 0.96	0.69 ± 1.10	0.918
Coronal tibial component (°) ^c^	0.21 ± 1.54	0.19 ± 1.06	−0.07 ± 0.68	0.580
Sagittal femoral component (°) ^d^	2.46 ± 2.17	1.94 ± 2.07	2.31 ± 2.53	0.057
Sagittal tibial component (°) ^d^	3.72 ± 2.42	0.86 ± 0.82	0.84 ± 0.77	< 0.001
Femoral component rotation (°) ^e^	−2.11 ± 1.58	1.62 ± 1.12	1.30 ± 0.83	< 0.001

^a^ TKA, total knee arthroplasty; HKA, hip–knee–ankle

^b^ One-way analysis of variance for continuous variables

^c^ A positive value denotes varus alignment

^d^ A positive value denotes extension alignment

^e^ A positive value denotes external rotation

**Table 3:** Proportion of outliers in the three groups

Variable	Conventional TKA (n = 50)	Robot-arm assisted TKA (n = 50)		p-value ^b^
		Initial stage	Proficiency stage	
HKA angle (°)	10/50 (20%)	1/18 (6%)	1/32 (3%)	0.048
Coronal femoral component (°)	9/50 (18%)	0/18 (0%)	1/32 (3%)	0.037
Coronal tibial component (°)	3/50 (6%)	0/18 (0%)	0/32 (0%)	0.290
Sagittal femoral component (°)	7/50 (14%)	2/18 (11%)	2/32 (6%)	0.500
Sagittal tibial component (°)	11/50 (22%)	2/18 (11%)	2/32 (6%)	0.007
Femoral component rotation (°)	6/50 (12%)	0/18 (0%)	0/32 (0%)	0.043

^a^ TKA, total knee arthroplasty; HKA, hip–knee–ankle

^b^ Pearson’s chi-square test or Fisher’s exact test for categorical variables

Groups I and P had fewer outliers in terms of the HKA angle (group C: 20%, group I: 6%, and group P: 3%; *p* = 0.048), femoral component coronal position (group C: 18%, group I: 0%, and group P: 3%; *p* = 0.037), axial position (group C: 12% and groups I and P: no outlier; *p* < 0.043), tibial component sagittal position (group C: 22%, group I: 11%, and group P: 6%; *p* = 0.007) than those in group C, and the differences between groups I and P were not significant (Table 3). The intra- and inter-observer measurement reliabilities were excellent for all parameters (ICC > 0.8, range: 0.83–0.91).

## Discussion

The primary findings of this study were as follows: (1) r-TKA was associated with a learning curve for operative time in 18 cases, and (2) irrespective of the learning curve, r-TKA had a lower outlier rate in terms of lower limb alignment, femoral component coronal position, axial position, and tibial component sagittal position. To the best of our knowledge, this is the first clinical study to analyze the learning curve for r-TKA and compare the implant positions between r-TKA and c-TKA according to the learning curve in Asian patients. As mentioned above, r-TKA demonstrated significant advantages for obtaining accurate limb alignment and implant position in the Asian population, given that conventional jigs and guides are susceptible to malpositioning due to the unique anatomical features of the Asian population.

In this study, the operative time was significantly shorter in groups C and P than that in group I, and no significant difference was observed between groups C and P. This result is consistent with those of previous studies, which reported that most surgeons record temporary prolonged operative time due to inexperience with the surgical technique in the initial phase. [22, 23] However, past the inflection point in the proficiency phase, the operative time was significantly shortened compared to that in the initial phase, and no significant difference was observed with c-TKA [24]. Although additional time is required for array fixation and bone registration in r-TKA, this may be the result of offsetting the additional time by a simple and accurate bone resection procedure without applying a cutting jig using the pre-cutting gap balancing technique. Two previous studies that employed CUSUM analysis reported that the number of cases required to attain the learning curve of operative time ranged from 7 to 11 cases, similar to our findings [17, 25]. However, Vermue et al. [18] found that the learning curve of operative time was longer, with a wider range of 11 to 43 cases. Since these results were recorded with relatively low-volume surgeons, they seem to have been influenced by the proficiency of each surgeon. Despite the late inflection point, these studies also showed no significant difference in the operative time compared to c-TKA in the proficiency phase.

In this study, r-TKA demonstrated fewer outliers in the lower limb alignment and femoral component coronal position than that with c-TKA. Achieving proper lower limb alignment in the coronal plane is essential for successful TKA [26]. A neutral mechanical axis permits even contact force on the joint and maintains adequate ligament tension [27]. Thus, failure to restore the neutral mechanical axis leads to unfavorable clinical outcomes and prosthesis failure. However, several studies have reported a considerable proportion of outliers exceeding 3° from the neutral mechanical axis in c-TKA [12, 28, 29]. For the distal femoral cutting procedure, the system of IM referencing has generally been adopted in c-TKA, which could be influenced by the individual anatomic factors of the femur [30]. Thus, patients with femoral deformities are vulnerable to inaccurate implantation. In particular, lateral bowing of the femoral shaft is common, with a high prevalence of up to 88% in Asian populations with severe OA. This causes varus orientation of the femoral component and functional disability [31–34]. In our study, no significant differences were observed in the mean values of both the coronal femoral and coronal tibial components. However, a difference in the outlier rates in the lower-limb alignment and the femoral component coronal position was identified between c-TKA and r-TKA. Therefore, femoral component malposition may contribute more to the lower limb alignment error than tibial component malposition. This difference may be attributed to the anatomical characteristics of the East Asian population in which lateral bowing of the femoral shaft is relatively common. We believe that r-TKA is advantageous for obtaining the planned coronal implant position and that better clinical results may be possible with better accuracy.

In this study, r-TKA demonstrated fewer outliers in the tibial component sagittal position than that with c-TKA. The posterior tibial slope is associated with the flexion gap, joint stability, and posterior femoral rollback, which further affect deep flexion and functional outcomes [35–38]. It is an especially important factor that can greatly affect patient satisfaction when considering the Asian lifestyle that favors a high knee flexion. Although previous studies demonstrated similar clinical results between the EM and IM techniques, the former was preferred because of concerns regarding the complications asscoiated with the use of IM rods, such as thromboembolism and intraoperative tibial fracture [39, 40]. However, placing an EM tibial cutting guide at the desired inclination is difficult. Generally, when the anterior border of the tibial crest is used as reference, the EM rod is manually matched to the mechanical axis of the tibia. However, this depends entirely on the operator’s sense and experience. Therefore, inter-individual consistency cannot be guaranteed. Previous studies have suggested that the anterior border of the tibia exhibited an inclination of approximately 2–5° to the mechanical axis. This suggests that such an error can occur if the posterior slope is set accordingly. In addition, the method of aligning the rod parallel to the longitudinal axis of the fibula is not highly accurate. The error may be wide, depending on the presence of tibial bowing and the thickness of soft tissues [41–43]. In contrast to c-TKA, the desired value is achieved accurately in the sagittal plane with the mechanical axis obtained from the preoperative CT scan in r-TKA. Therefore, r-TKA is also advantageous for obtaining the planned implant position in the sagittal plane.

In this study, r-TKA had fewer outliers in the femoral component axial position than that with c-TKA. Malrotation of the femoral component can cause patellofemoral maltracking after TKA. It can lead to undesirable complications, such as anterior knee pain, instability, loosening, and fracture. This may also induce patient dissatisfaction and early prosthesis failure [44–47]. However, confirming the rotational alignment intraoperatively can be difficult. For the measurement of FCR alignment, the PCA, TEA, and Whiteside method with the AP axis were introduced. Although the anatomical TEA and sTEA are well-known landmarks, they are difficult to visualize because they are surrounded by structures such as the collateral ligament and soft tissues [48]. Hence, several studies have emphasized the low inter-individual consistency in determining TEA [49–51]. In contrast, TEA can be accurately defined using preoperative CT scans with an error of < 1 mm in r-TKA. Thus, we believe that r-TKA is also advantageous for obtaining the planned femoral implant position in the axial plane.

This study had some limitations. First, it focused only on the radiographic outcomes. Therefore, the manner in which accurate lower-limb alignment and implant position affect the outcomes remains unclear. Further studies are required to confirm these findings. In addition, since the study was conducted with only one high-volume surgeon, its applicability to less experienced surgeons is unknown.

## Conclusion

Although r-TKA had a learning curve for operative time in 18 cases, the operative time was not different between r-TKA and c-TKA after the learning curve. Moreover, surgeons could expect more accurate and reproducible lower-limb alignment and implant position with r-TKA in Asian patients, regardless of the learning curve.

## Data Availability

The data presented in this study are available on request from the corresponding author. The data are not publicly available.

## Abbreviations

r-TKA: Robot-assisted total knee arthroplasty
c-TKA: Conventional total knee arthroplasty
OA: Knee osteoarthritis
TKA: Total knee arthroplasty
CT: Computed tomography
IM: Intramedullary
EM: Extramedullary
FCR: Femoral component rotation
POD: Postoperative day
HKA: Hip–knee–ankle
AP: Anteroposterior
CFA: Coronal femoral alignment
CTA: Coronal tibial alignment
SFA: Sagittal femoral alignment
STA: Sagittal tibial alignment
sTEA: Surgical transepicondylar axis
PCA: Posterior condylar axis
CUSUM: Cumulative summation

## Notes

The original version of this article was revised: the authors identified a significant typographical error in Figure 1 (incorrect labeling of therapeutic groups). Instead of labeling one as the r-group, they marked both as the c-group.
