# Correction: Learning curve of robot-assisted total knee arthroplasty and its effects on implant position in asian patients: a prospective study

**DOI:** 10.1186/s12891-023-06645-x

**Published:** 2023-06-21

**Authors:** Ho Jung Jung, Min Wook Kang, Jong Hwa Lee, Joong Il Kim

**Affiliations:** Department of Orthopedic Surgery, Kangnam Sacred Heart Hospital, HallymUniversity College of Medicine, Seoul, Korea

**Correction: BMC Musculoskelet Disord 24, 332 (2023)**

**https://doi.org/10.1186/s12891-023-06422-w**

Following publication of the original article [1], the authors identified a significant typographical error in Fig. 1 (incorrect labeling of therapeutic groups). Instead of labeling one as the r-group, they marked both as the c-group. The correct figure is given below.

**Fig. 1:**
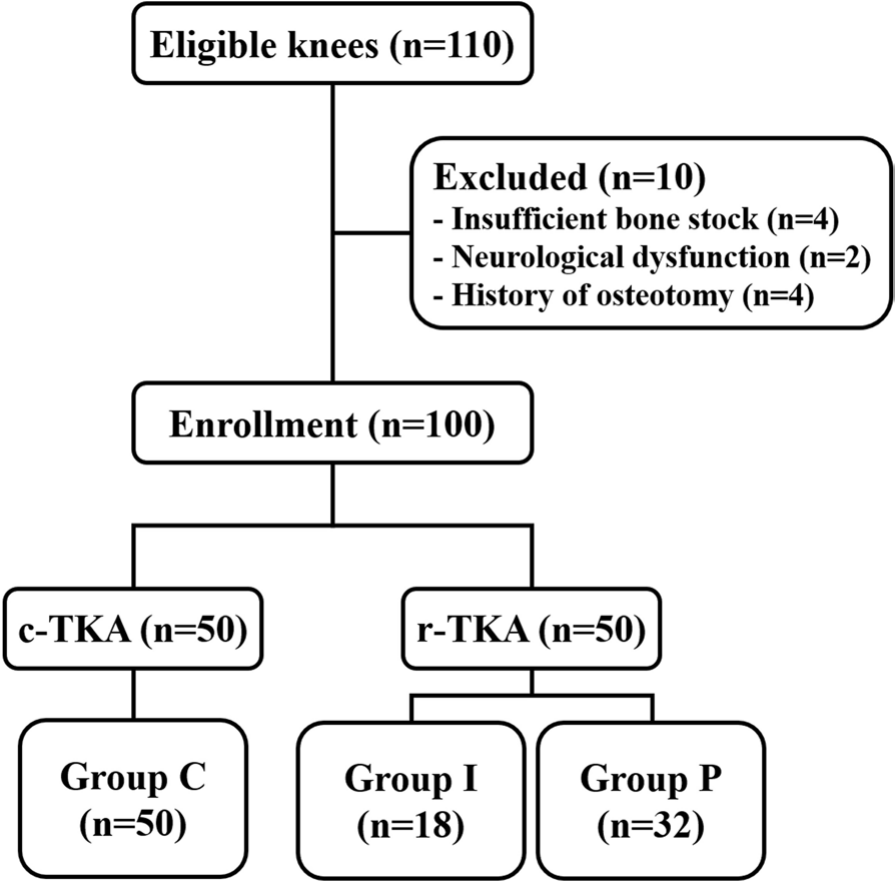
Flow diagram of patient enrollment. *c-TKA, conventional total knee arthroplasty; r-TKA, robot-assisted total knee arthroplasty; group C, conventional total knee arthroplasty group; group I, initial phase group; group P, proficiency phase group

The original article [1] has been corrected.
