# Correction: Learning curve of robot-assisted total knee arthroplasty and its effects on implant position in asian patients: a prospective study

**DOI:** 10.1186/s12891-023-06852-6

**Published:** 2023-10-02

**Authors:** Ho Jung Jung, Min Wook Kang, Jong Hwa Lee, Joong Il Kim

**Affiliations:** Department of Orthopedic Surgery, Kangnam Sacred Heart Hospital, Hallym University College of Medicine, Seoul, Korea

**Correction:** ***BMC Musculoskelet Disord*** **24, 332 (2023)**

10.1186/s12891-023-06422-w

Following publication of the original article [1], the authors identified the following minor typographical errors in Tables 1 and 3, which are not significant regarding result.

Table 1:

K-L grade IV (%) -> K-L grade (III/IV)

Table 3:

HKA angle of proficiency stage: 1/32 (6%) -> 1/32 (3%)

The original article [1] has been corrected.
